# Study on the Characteristics of Urban Residents’ Commuting Behavior and Influencing Factors from the Perspective of Resilience Theory: Theoretical Construction and Empirical Analysis from Nanjing, China

**DOI:** 10.3390/ijerph17051475

**Published:** 2020-02-25

**Authors:** Honghu Sun, Feng Zhen, Yupei Jiang

**Affiliations:** 1 School of Architecture and Urban Planning, Nanjing University, Nanjing 210093, China; shhupup@163.com (H.S.); jiangyupeidl@126.com (Y.J.); 2 Provincial Engineering Laboratory of Smart City Design Simulation & Visualization, Jiangsu, Nanjing University, Nanjing 210093, China

**Keywords:** commuting behavior, resilience theory, characteristics, influencing factors, built environment, China

## Abstract

In the transitional period of China’s urbanization, commuting problems and demands are diversified and multi-level, so commuting research topics, viewpoints, and analysis paths should be organically combined to dynamically adapt to the complex commuting contradictions. Based on this, this paper introduces the resilience theory to improve the research paradigm of commuting behavior. Taking Nanjing, China as a case study, with the help of the survey data of commuting behavior of typical communities, this paper provides an empirical analysis of the characteristics and influencing factors of urban residents’ commuting behavior from the perspective of resilience theory. The results show that: (1) in the face of commuting pressure, to a large extent, most commuters can still obtain commuting adaptability and a medium level or higher of commuting resilience; and (2) personal attributes, living and employment environment, and commuting environment all have significant heterogeneity effects on commuting pressure, commuting adaptability, and commuting resilience. From the perspective of resilience theory, the means of regulating commuting conflicts are flexible, which can not only directly reduce commuting pressure or optimize commuting adaptability, but also improve commuting resilience according to the specific commuting scenarios constructed by commuting pressure and adaptability. On the whole, the principles of comprehensive improvement, on-demand supply, and dynamic adjustment should be followed.

## 1. Introduction

Commuting is a daily travel behavior caused by the spatial separation between a person’s place of residence and place of employment. It can reflect the quality of daily life and the efficiency and fairness of urban space and, as a result, is an important research topic in urban planning, urban geography, and urban sociology [1,2,3]. In the West, a great deal of research has been done on commuting theory and models [4,5,6,7], factors affecting commuting behavior [8,9,10], commuting and spatial imbalance [11,12,13], commuting effects [14,15,16], and other topics. At present, the research focus has shifted from simple facilities construction to residents’ travel demand management [17,18,19]. Based on the western research experience, Chinese scholars have analyzed daily commuting in large cities, such as Beijing [20,21], Guangzhou [22,23], and Shanghai [24,25], and identified similarities and differences in commuting laws during the different backgrounds and stages of urban development in China and the West. Breakthroughs have also been made in new technology methods and applications [26,27,28]. However, in terms of the current process of urbanization in China, the new and old problems and demands of commuting faced by residents in the period of urban development and transformation are often intertwined and overlapped, with characteristics of diversity, multi-level, and complex dynamic evolution. Although relevant research in western academic circles can provide experience for China’s commuting issues, due to the different background and stages of urbanization development, it is difficult to directly apply this research to the Chinese case. On the whole, the existing commuting research paradigm is mainly a single research topic, an indirect research perspective, and a linear analysis path. Due to the lack of integrated research on commuting contradictions in different commuting scenarios at the micro-individual scale, it is often difficult to comprehensively and specifically resolve commuting contradictions, and consider commuting efficiency, effects, and needs at the same time. There is an urgent need for theoretical research integration and innovation to analyze the current background and stage of China’s urbanization in a more clear, comprehensive, and timely manner.

Therefore, keeping abreast of the contradiction between people’s growing needs for a better life and the unbalanced and inadequate development during the transition period of China’s urbanization development [29], based on the dilemma of theoretical integration and practical regulation of commuting research, this study focuses on the commuting ability of individuals in the face of their commuting situations. First, it attempts to integrate the research topics of commuting opportunities and constraints; commuting efficiency and fairness; and commuting demand management and effective supply into the commuting contradiction. Second, by introducing the resilience theory and drawing on its theoretical advantages of emphasizing interaction and dynamics from two dimensions of absolute ability and relative ability, it constructs a resilience perspective that directly reflects personal commuting ability. Third, the concepts of “commuting pressure”, “commuting adaptability”, and “commuting resilience” are put forward. The interaction of “commuting pressure and adaptability” is regarded as a contradictory unity to promote the dynamic evolution of commuting resilience, the different combinations of which are used to construct commuting scenarios to represent commuting resilience. Finally, with the help of a typical community residents’ commuting behavior survey, this paper makes an empirical analysis of the characteristics and influencing factors of urban residents’ commuting behavior from the perspective of resilience theory. This study attempts to provide a new and useful exploration for the theoretical and practical research of commuting behavior.

## 2. Construction and Discrimination of Concepts

### 2.1. Construction of Concepts Related to Commuting Resilience

At its core, the concept of “resilience” is the ability of a system to resist, absorb, transform, and adapt to interference, impact, or uncertainty without changing its basic conditions [30,31]. Its theoretical development began with a focus on engineering resilience in a single steady state, and then the theory of ecological resilience with multiple equilibriums developed rapidly. At present, it is further improved to reveal the dynamic evolution resilience of self-organization, self-learning, and adaptive characteristics of a social-ecological composite system [32,33,34]. “Resilience” is generated and characterized by the interaction of various disturbance forces and adaptive forces. It emphasizes that risks, pressures, and shocks are unavoidable, which is difficult to fully control and has dynamic changes. Simultaneously, in a resilient system, disturbances can be actively dealt with and effectively adapted, resilience can be achieved by turning challenges into opportunities. Due to the innovation of the resilience theory in the research paradigms of dynamic adjustment, multidimensional interaction, and active response, it has achieved a great degree of development and application in the fields of ecology, global change, urban planning, and urban geography [35,36,37,38]. 

As far as commuting behavior is concerned, commuting is the process of travel between the place of residence and the place of work whereby the process is inevitable and not the purpose of the activity itself; In addition, commuting consumes time and may have negative effects such as personal financial expenses, health risks, and environmental burdens [14,39,40,41]. Obviously, the incidental and costly nature of commuting has the effect of commuting pressure; accordingly, commuters will make suitable commuting arrangements—called commuting adaptability—based on personal conditions and the external environment to adapt to the commuting pressure they face. Then, according to resilience theory, this kind of relative commuting ability demonstrated by adapting to commuting pressure can be called commuting resilience.

### 2.2. Discrimination of Concepts Similar to Commuting Resilience

The concepts of commuting resilience, commuting flexibility and commuting accessibility are similar but have different connotations. Commuting resilience characterizes a comprehensive commuting ability under a commuting scenario constructed by commuting pressure and adaptability and has a positive effect. Commuting flexibility refers to the variability of commuting behavior, such as the variability of commute distance, time, path, and mode [42,43], however, this variability is only a description of the state. Although it brings flexibility to commuting demand management, it is difficult to determine whether the flexibility of commuting has a positive effect on the commuter without knowing the specific commuting scenario, making it difficult to upgrade commuting demand management to individual-oriented commuting demand services. Both commuting resilience and accessibility reflect commuting ability, but their specific concepts have different connotations and extensions. Commuting resilience expresses the relative commuting ability of specific scenarios, and also pays attention to absolute commuting pressure and commuting adaptability, which can fully reflect commuting ability; commuting accessibility indicates the absolute convenience of commuting [44,45], therefore, it cannot identify the relative commuting ability of individuals.

## 3. Data sources and Research Methods

### 3.1. Overview of the Study Area

**Figure 1:**
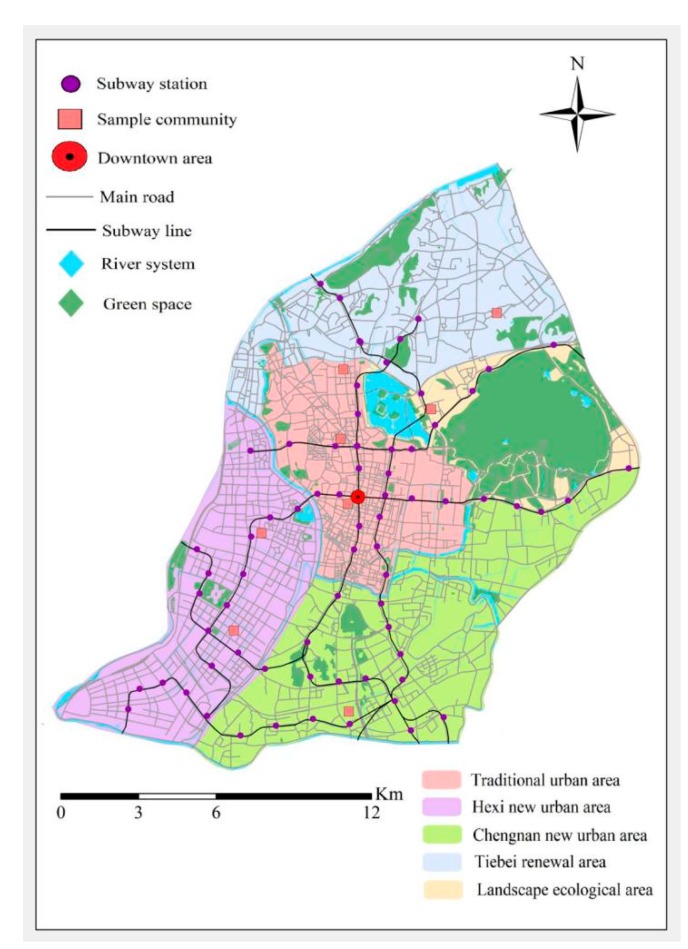
The study area and the location of sample community.

Nanjing is an important central city in the east of China. In 2018, the resident population was 8.46 million, and the urbanization rate was 82.5%. However, Nanjing’s excess commuting index reached about 0.55, the average one-way commuting distance reached 8.9 km, and the average one-way commuting time reached 42.3 min; there was a relatively serious imbalance between occupation and residence, which ranks medium among major cities in China [46]. This study takes the traditional main city of Nanjing as the sample collection area for the survey of commuting activities (Figure 1). This area has the most concentrated population, the most complex and diverse urban space, and the most frequent commuting problems. Therefore, this case study in Nanjing is typical and complete.

### 3.2. Data Sources and Basic Characteristics

The data are derived mainly from the “Nanjing Residents’ Physical Activity and Health Status Survey” conducted by the author’s research team from December 2017 to January 2018. Residents’ daily physical activity information was collected through home interviews, and 503 samples with commuting behaviors were taken from them, of which 468 samples contained complete information needed for the study. The survey content involves personal socio-economic attributes, physical status, commuting time and space characteristics, and occupational and residential locations of commuters. In addition, the urban built environment data are obtained through the network open platform, which includes information about real estate, recruitment, road networks, bus stations, subway stations, and other urban markers.

According to the characteristics of personal attributes (Table 1), among the residents who commute, the number of men is slightly higher than that of women; the large majority have a college education; and most of them are under 50 years old, married, live locally, and live in family groups of three or more. The majority of commuters have a normal body type, with a certain proportion of them being overweight. Their occupations are mainly enterprise employees, and government employees account for a certain proportion. Their personal income and family income are relatively balanced. More than half of the families own private cars.

In terms of basic commuting characteristics (Table 2), the proportion of respondents who commute by private car is the highest, the proportions of those who commute by subway and walking are close behind, and the proportion of respondents who commute by company shuttle is the lowest. On the whole, modes of commuting are mainly green travel represented by non-motorized transport and public transport. Second, the average one-way commuting distance using subway, public bus, private car, and company shuttle as commuting methods is greater than the average level in Nanjing (8.9 km), while the average one-way commuting distance of walking, bicycle, and electrical motorcycle are smaller than the average level in Nanjing. It can be seen that the commuting distance is closely related to the commuting mode, mainly manifested in the difference of commuting distance between motorized travel and non-motorized travel. Third, except for the company shuttle, the average one-way commuting time of other commuting methods is less than the average level in Nanjing (42.3 km). Among them, the average one-way commuting time of subway and public bus is close to the average one-way commuting time in Nanjing, and the average one-way commuting time of walking, bicycle, and electrical motorcycle is much lower than the average level in Nanjing. It can be seen that there is a similar law to the average one-way commuting distance.

### 3.3. Research Ideas and Methods

**Figure 2:**
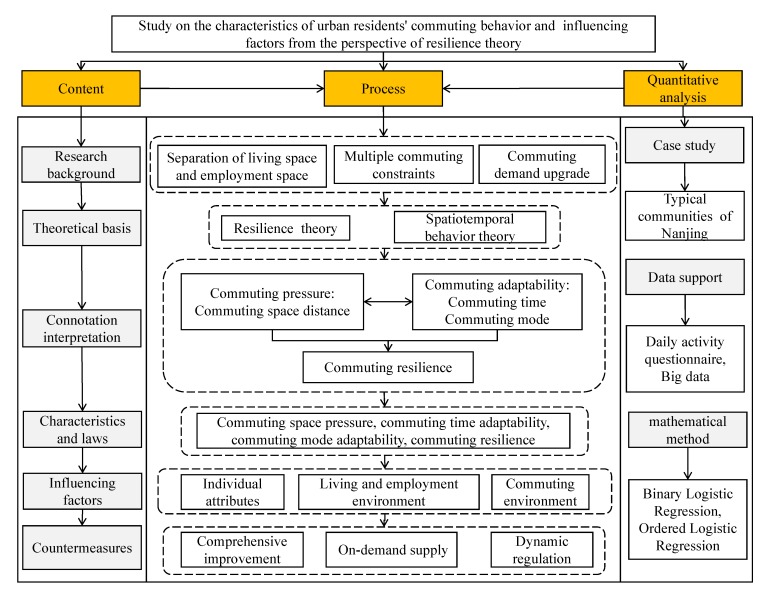
An analytical framework of urban residents’ commuting behavior characteristics and influencing factors from the perspective of resilience theory.

During the period of China’s urban development and transformation, there are some contradictions of the separation of working and living space, the diversity of commuting problems, and the upgrading of commuting demand. The resilience theory regards this contradiction as a unity of opposites between commuting pressure and adaptability. The spatiotemporal characteristics of commuting behavior are the result of the dynamic evolution and interactive transformation of commuting pressure and adaptation. By constructing the indices of commuting pressure, adaptability, and resilience, we can see the connotation of commuting contradiction and interpret the actual commuting ability of individuals at the micro level. Based on this, the study takes Nanjing as a typical case area to investigate the commuting behavior of urban residents, identify the commuting characteristics and influencing factors from the perspective of resilience theory, and refine the optimization concept of comprehensive improvement, on-demand supply, and dynamic regulation. The basic analysis framework is shown in Figure 2.

From the perspective of resilience theory, the analysis of commuting characteristics of urban commuters first needs to determine how to characterize commuting pressure and commuting adaptability. For commuting behavior, the direct pressure source is undoubtedly the space restriction and opportunity of the commuting distance. Therefore, commuting distance can be used to represent commuting pressure. Commuting time and mode are the behavior mapping of commuters after making adaptive decisions on commuting distance, in which commuting time reflects the adaptive cost of commuting in the time dimension, while commuting mode can better reflect commuting in terms of economic cost, health promotion, environmental protection, and other dimensions. Thus, commuting adaptability can be considered in terms of two aspects: commuting time and mode. According to the criteria of whether the commuting distance is greater than the average commuting distance in Nanjing, whether the commuting time is less than the average commuting time in Nanjing, and whether the commuting mode is green or not, commuting space pressure, commuting time adaptability and commuting mode adaptability are distinguished. Different interval combinations of commuting pressure and adaptability are regarded as different commuting scenarios to judge commuting resilience level. The judgment matrix of commuting characteristics from the perspective of resilience theory is as follows (Table 3). In particular, there are two levels of commuting pressure and commuting adaptability: yes or no, whose combination of levels will constitute four levels of commuting resilience: none, low, medium, high. The basic principle of constructing the commuting resilience level is that the larger the positive gap between the commuting adaptability level and the commuting pressure level, the higher the resilience level.

Considering that commuting is the result of separation of living space and employment space, previous studies have found that commuting is closely related to individual attributes and urban built environment. In the framework of urban planning or urban geography, more attention is paid to urban material or social space, so it is further divided into working living environment and commuting environment from the perspective of comprehensive and targeted regulation of urban built environment. Finally, the index system of independent variables is constructed from three dimensions: personal attributes, occupation living environment, and commuting environment. Moreover, because different commuters face different commute problems and needs, commute pressure, commute time adaptability, commute mode adaptability, and commute toughness all need to be analyzed as dependent variables (Table 4).

In terms of the data structure characteristics of dependent variables, commuting pressure, commuting time adaptability, and commuting mode adaptability are divided into "yes" and "no," which are typical binary classification variables, so binary logistic regression can be used to analyze the influencing factors [47]. Commuting toughness is divided into different orders according to the different interval combination of commuting pressure and adaptability, which makes it suitable to use multiple ordered logistic regression to analyze the influencing factors [48].

## 4. Results

### 4.1. Characteristics of Commuting Behavior from the Perspective of Resilience Theory

As shown in Table 5, more than half of the respondents have commuting space pressure, slightly more than the number without commuting space pressure; at the same time, the proportion of commuters with commuting time adaptability and commuting mode adaptability is similar, and is far larger than the number without commuting adaptability. According to the judgment of the commuting situation based on the combination of different intervals of commuting pressure and adaptability, commuting resilience shows three grades. Among them, the number of commuters with medium-level resilience has an absolute advantage, followed by the number of commuters with high-level resilience; the number of commuters with low-level resilience is the lowest. Although nearly half of the commuters face the pressure of absolute commuting space, most of them have better absolute commuting adaptability as well as a medium or higher level of commuting resilience. 

### 4.2. Influencing Factors of Commuting Behavior from the Perspective of Resilience Theory

As shown in Table 6, influential factor analysis for commuting space pressure, commuting time adaptability, commuting mode adaptability based on the binary logistic regression, and influential factor analysis for commuting resilience based on the multiple ordered logistic regression all have good fitting effects.

#### 4.2.1. Influencing Factors of Commuting Space Pressure

As far as commuting space pressure is concerned, the indicators that affect it include mainly type of jobs, BMI, household registration, marital status, and household monthly income in the dimension of personal attributes; housing prices and diversity of infrastructure in the place of residence in the dimension of living and employment environment; and subway station density in the place of residence, distance from the place of residence to the city center, bus station density in the place of employment, and distance from the place of employment to the city center in the dimension of commuting environment. Specifically, in terms of personal attributes compared with general service personnel, civil servants and enterprise employees have a higher probability of commuting space pressure, probably because their work system is more fixed, while general service personnel have a wider employment demand and the commuting system is more flexible so they can get employment nearby, thus reducing the commuting space pressure. Compared with commuters with local household registration, commuters without local household registration are less likely to have commuting pressure, which may be due to their more flexible choice of residence and employment place. To some extent, unmarried commuters are less likely to have commuting pressure than married commuters, which may be because married commuters need to consider more aspects of commuting decision-making, especially family affairs. Compared with high-income families, commuters in the lower and middle-income families have a lower probability of commuting space pressure. Obviously, there are a few opportunities for the whole family to work nearby and have a high salary. In terms of living and employment environment, the higher the housing prices, the greater the probability of having commuting space pressure. This may be because the higher the housing prices, the better the built environment and personal ability to support commuting, and the more likely that long-distance commuting is promoted. To some extent, the diversity of residential facilities will reduce the probability of commuting space pressure, which may be because the diversity of residential facilities will improve commuting efficiency on the one hand, and reduce the interference of non-commuting travel on commuting travel on the other hand. In terms of commuting environment, the subway station density of the residence has a significant negative impact on the commuting pressure probability, which may be because the subway traffic volume is large and the commuting time is more secure, which will gather employment resources. The distance from the residence to the city center also has a significant impact on reducing the pressure of commuting space, which may be because the commuters who live far away from the city center tend to work nearby their residences. The density of public transport stations in employment places can promote the probability of commuting space pressure. The higher the density of bus stations in employment places, the more convenient commuting will be, which will promote long-distance commuting. At a significant level, the distance from the place of employment to the city center has the effect of increasing the probability of commuting space pressure. At present, Nanjing’s urban spatial structure still has significant single-core characteristics. The farther the employment location is from the city center, then, the worse the transportation location, so the commuting space pressure is higher.

#### 4.2.2. Influencing Factors of Commuting Time Adaptability

There are a few factors affecting the adaptability of commuting time: personal attributes such as personal monthly income, the number of private cars owned by a family, and the distance from the employment center to the city center related to the commuting environment. Employment and living conditions have little effect on the adaptability of commuting time. In terms of personal monthly income, compared with the highest personal monthly income range, the probability of commuting time adaptability in other income ranges is significantly lower, but the difference between other income ranges is not significant. This may be because the high-income groups pursue higher efficiency of commuting time and have more freedom and flexibility in the use of commuting time. Compared with households with two or more private cars, the probability of commuting time adaptability of commuters with one private car and no private car decreases in turn, indicating that access to a private car improves commuting time adaptability, which may be due to the higher time efficiency of private car travel within a certain commuting distance. The living and employment environment has no effect on the adaptability of commute time. In terms of commuting environment, only the distance from the place of employment to the city center has a negative impact on a significant level. On the one hand, the choice of working and living environment is not based on the consideration of commuting time adaptation. On the other hand, due to the relatively balanced distribution of commuting facilities, the impact of employment location on commuting time adaptation is more prominent.

#### 4.2.3. Influencing Factors of Commuting Mode Adaptability

In terms of the adaptability of commuting mode, there are many influencing factors. The main indicators relating to personal attributes are: occupation type, BMI, gender, age, household registration, personal monthly income, and number of private cars. The influencing factors relating to the living and employment environment are: housing price, recruitment volume, and diversity of infrastructure in the place of residence, as well as housing prices and recruitment volume in the place of employment. Indicators of the adaptability of commuting mode relating to the commuting environment are: subway station density and bus station density in the place of residence, distance from the place of residence to the city center, and subway station density and bus station density in the place of employment. Specifically, compared with general service personnel, the probability of adaptability of commuting mode of enterprise employees and self-employed laborers is lower, mainly because their commuting distance is generally longer and they are willing and have the strength to pursue commuting time efficiency. The commuters with thin body shape have a lower probability of adapting to commuting mode than those who are obese. It can be assumed that obese commuters prefer to travel green, rather than that non-green travel leads to obesity. Additionally, women prefer green commuting to men, which may be related to social roles and commuting preferences. Compared with commuters aged 50 and older, commuters aged 30 and younger and in the 40–50 age range have a lower probability of adaptability to commuting mode, which may be related to commuting preference or economic strength. Compared with local household registration, commuters with non-local household registration have a higher probability of adaptability to commuting mode at a certain significant level, but this adaptability may be based on low living and employment level. Compared with the group with the highest monthly personal income, the adaptability probability of commuting mode of the relatively high income group and the lowest income group increases in turn at a certain significant level. It can be seen that economic strength only affects the commuting mode of some groups; for commuters with two or more private cars, commuters with no private car and one private car have a much higher probability of adaptability to commuting mode. The more private cars they own, the more likely they are to commute by private car rather than travel green. In terms of living and employment environment, the housing prices in the residential area have a negative impact on the adaptability of commuting mode at a significant level. To a large extent, the housing prices represent the comprehensive quality of the living environment. Commuters living in high housing price areas may pay more attention to the convenience and comfort of commuting than the economic cost and energy consumption. At this time, the probability of commuting by private car may be higher. The level of recruitment in residential areas has a positive impact on the adaptability of commuting mode at a more significant level. Obviously, if there are employment opportunities near the residential area, the commuters prefer to work nearby if other conditions remain unchanged. The diversity of residential facilities has a significant positive impact. The improvement of residential facilities will comprehensively improve the adaptability of commuting mode and then promote green travel. Housing prices and recruitment level have a significant positive impact on the adaptability of commuting at a certain significant level. On one hand, the higher the housing prices in the employment area, the better the comprehensive quality of the built environment; on the other hand, the more the number of employment places, the more the employment opportunities. Obviously, a good living and employment environment in the employment area will promote the adaptability of commuting time. In terms of commuting environment, the density of metro stations in residential areas has a significant positive impact, while the density of bus stations in residential areas has a significant negative impact. It may be because of the convenient, stable, and large transportation capacity of the subway. In contrast, bus commuting does not have these advantages. The high density of bus stops may face the pressure of higher commuting demand, which leads to the negative impacts of low commuting efficiency, long wait times, and overcrowding. The distance from the residential location to the city center has a significant positive impact. It may be that the inconvenient location of the residence will cause commuters to get employment nearby, which indirectly promotes green commuting. The bus station density in the employment area has a positive impact at a certain level. Obviously, the high density of public stations in employment areas will increase the chances of commuters using the bus for green commuting.

#### 4.2.4. Influencing Factors of Commuting Resilience

As far as commuting resilience is concerned, under the commuting scenario that considers the combination of commuting space pressure, commuting time adaptability, and commuting mode adaptability, the influencing factors relating to personal attributes include mainly BMI, gender, the number of private cars owned by the family; the influencing factors relating to the living and employment environment are the diversity of residential facilities and the housing prices in the place of employment; and the influencing factors relating to commuting environment are the density of bus stops in the place of residence and the density of bus stops in the place of employment. In terms of personal attributes, compared with obese body types, commuters with thin, normal, and overweight body shape have negative effects on commuting resilience but weakens in order. Compared with men, women have a significantly higher probability of commuting resilience, which is mainly related to women’s commuting time and mode. Compared with commuters who have two or more private cars in their families, commuters who do not have a private car and commuters who own one private car have a higher probability of commuting resilience, but decrease in turn. This shows that, in terms of contribution to commuting resilience, the overall adaptability of commuting mode is higher than the adaptability of commuting time. In terms of living and employment environment, the diversity of residential facilities and the price of housing in the place of employment have a positive impact on the resilience of commuting at a significant level. This may be because the improvement of residential facilities will not only guarantee commuting activities, but also reduce the interference of other living activities on commuting activities. Furthermore, the higher price of housing in the place of employment comprehensively reflects the better level of infrastructure and traffic location of the place of employment, which can provide effective support in commuting efficiency and green commuting. In terms of commuting environment, bus station density in the place of residence and in the place of employment has certain negative and positive effects, respectively. In residential areas, the quantity of bus stops has no effect and may even have a negative effect on the improvement of commuting resilience. This may be because where demand for public transportation commuting in residential areas has been upgraded, bus travel can no longer match this demand, but at the same time, the demand for bus commuting in employment areas has not reached saturation.

## 5. Discussion and Conclusions

In the transition period of China’s urbanization development, diverse and multi-layered commuting problems and demands are interwoven and superimposed so that the organic integration of commuting research topics, perspectives, and analysis paths is required to dynamically adapt to the complex commuting contradictions. Based on this, resilience theory is introduced to construct a new paradigm of commuting behavior research. This paper puts forward the concepts of “commuting pressure”, “commuting adaptability”, and “commuting resilience”, and regards “commuting pressure and adaptability” as the contradictory unity to promote the dynamic evolution of commuting resilience. In this way, research topics such as commuting opportunities and constraints, commuting efficiency and fairness, commuting demand management, and effective supply are unified into the overall concern of commuting contradictions, the research perspectives of problem-based, objective-based and effect-based are transferred to the perspective of resilience of improving the actual commuting ability of individuals, and the one-way, static, and linear analysis path is promoted to the scenario analysis composed of commuting pressure and adaptability. Taking Nanjing, China as a case study, this paper also makes an empirical analysis on the characteristics and influencing factors of urban residents’ commuting behavior from the perspective of resilience theory. Based on this study, the following conclusions can be drawn.

(1) In the face of the specific commuting situation formed by the combination of commuting pressure and adaptability, although more than half of the respondents have the pressure of commuting space, most of them have the adaptability of commuting time and commuting mode. Also, most respondents have a medium or above level of commuting resilience, which is mainly at the medium level. This is consistent with the fact that although most of the major cities in China have large-scale built-up areas, public transport of these cities are relatively developed and become the main mode of commuting, which has an effect that most commuters are adaptable to the time and mode of commuting when facing the pressure of commuting space. [46,49,50,51]. In fact, the commuters of Nanjing have a relatively balanced and sufficient commuting ability, and their diversified and multi-level needs, such as time efficiency, economic cost, and health effects, can be better met.

(2) Personal attributes, living and employment environment, and commuting environment have significant and different effects on commuting behavior from the perspective of resilience theory. The pressure of commuting space is affected mainly by occupation type, commuting facilities, and residence and employment location; the adaptability of commuting time is affected mainly by personal economic strength and employment location; the adaptability of commuting mode is influenced by a wide range of factors, mainly by social attributes such as individual occupation type, gender and age, as well as the living and employment environment, and commuting environment in residential areas; the resilience of commuting is affected mainly by non-economic factors such as BMI, gender, and commuting facilities. On the whole, considering the pressure of commuting space, the adaptability of commuting time, and the adaptability of commuting mode in a single dimension, their influencing factors and action directions are similar to most studies [52,53,54]. Among them, there are many directions to optimize the commuting space pressure and the commuting mode adaptability, but there are not many effective ways to improve the commuting time adaptability. In terms of commuting resilience, which reflects the interaction between commuting pressure and adaptability, it is more affected by physical activity habits reflected by non-economic factors and the facility environment.

(3) In the face of commuting contradictions, first, the object of regulating commuting behavior is flexible from the perspective of resilience theory. On the one hand, commuting pressure and commuting adaptability can be optimized at an absolute level to directly reduce commuting pressure or improve commuting adaptability. On the other hand, commuting resilience can also be improved based on the combination of commuting pressure and adaptability, with the optimization of relative commuting ability then realized at a lower cost. Second, because there are many factors influencing commuting behavior from the perspective of resilience theory, the means of regulation can be more diverse. More consideration should be given to the social differentiation of commuters to guide commuting behavior, and at the same time, the spatial organization, structure, and function of the built environment should be improved through commuting behavior. On the whole, the regulation strategy of commuting contradiction from the perspective of resilience theory needs to follow the principles of comprehensive improvement, on-demand supply, and dynamic adjustment. 

(4) In view of the diversity and multi-level of commuting problems and demands in the period of urban development and transformation, this paper puts forward a research paradigm based on the perspective of resilience theory, and makes an empirical analysis, which integrates the research theme, opens up the research perspective, enriches the research path, and provides a quantitative analysis. In addition, the core of resilience theory is how to promote development in contradiction interaction and then break through paradoxical constraints [55]. The study of commuting behavior from the perspective of resilience theory pays more attention to the problems and needs of commuters in commuting contradiction. Therefore, the concept of commuting demand service is injected into commuting demand management. Simultaneously, although the perspective of resilience theory is more integrated to meet the needs of practice, we only studied the objective state of the evolution and interaction of commuting contradictions and a simple construction of a commuting situation. The subjective content of commuting perception and the decision-making process were not considered in our study; thus, the mode, mechanism, and effect of the dynamic evolution of subjective and objective interaction need further analysis.

## Figures and Tables

**Table 1:** Basic personal attributes of commuters.

Personal Attributes	Type of Attribute	Percentage of Commuters (%)	Personal Attributes	Type of Attribute	Percentage of Commuters (%)
Gender	Male	53	Marriage	Married	76
Age	Female	47		Unmarried	24
	30 and below	33	Type of jobs	Civil servant	23
	30–40	34		Enterprise staff	59
	40–50	21		Self-employed laborer	3
	50 and above	12		General service staff	15
Household registration	Local registration	72	BMI	Thin	5
	Non-local registration	28		Normal	60
Education	High school degree and below	18		Slightly overweight	25
	Bachelor’s degree	65		Obese	10
	Master’s degree and above	17	Personal monthly income	4000 and below	18
Number of family members living together	1	11		4000–6000	22
	2	18		6000–8000	17
	3	43		8000–10,000	17
	4 and above	28		10,000–15,000	13
Household monthly income	8000 and below	19		15,000 and above	13
	8000–12,000	18	Number of cars owned by family	0	35
	12,000–16,000	19		1	51
	16,000–20,000	14		2 and above	14
	20,000–30,000	17			
	30,000 and above	13			

Notes: The unit of personal monthly income and household monthly income are Yuan, and 7 Yuan is roughly equivalent to 1 USD. BMI is Body Mass Index.

**Table 2:** Basic commuting characteristics.

Main Mode of Commuting	Percentage of Commuters (%)	Average One-Way Commuting Distance (km)	Average One-Way Commuting Time (min)
Walking	20	1.1	13.2
Bicycle	8	6	21.8
Electrical motorcycle	13	6.7	19.3
Subway	22	17.2	42
Public bus	11	15.1	40.3
Private car	23	16.6	31
Company shuttle	3	20.8	50.4

**Table 3:** The judgment matrix of commuting characteristics from the perspective of resilience theory.

Commuting Space Pressure	Commuting Time Adaptability	Commuting Mode Adaptability	Commuting Resilience
No (0)	Yes (1)	Yes (1)	Medium (2)
No (0)	Yes (1)	No (0)	Low (1)
No (0)	No (0)	Yes (1)	Low (1)
Yes (1)	Yes (1)	Yes (1)	High (3)
Yes (1)	Yes (1)	No (0)	Medium (2)
Yes (1)	No (0)	Yes (1)	Medium (2)
No (0)	No (0)	No (0)	None (0)
Yes (1)	No (0)	No (0)	Low (1)

**Table 4:** The influencing factors system of commuting characteristics from the perspective of resilience theory.

Dependent Variable	Independent Variable	
	Dimension	Index
Commuting space pressure	Personal attributes	Type of jobs, BMI (Body Mass Index), gender, age, household registration, education level, marital status, number of family members living together, personal monthly income, household monthly income, number of cars owned by family
Commuting time Adaptability	Living and employment environment	Housing price in the place of residence, recruitment volume in the place of residence, diversity of infrastructure in the place of residence, housing price in the place of employment, recruitment volume in the place of employment, diversity of infrastructure in the place of employment
Commuting mode adaptability	Commuting environment	Subway station density in the place of residence, bus station density in the place of residence, distance from the place of residence to the city center, subway station density in the place of employment, bus station density in the place of employment
Commuting resilience		

**Table 5:** Characteristics of commuting behavior from the perspective of resilience theory.

Characteristics of Commuting Behavior	Types of Commuting Behavior Characteristics	Percentage of Commuters (%)
Commuting space pressure	Yes	53
	No	47
Commuting time adaptability	Yes	79
	No	21
Commuting mode adaptability	Yes	77
	No	23
Commuting resilience	Low	8
	Medium	74
	High	18

**Table 6:** Analysis of influencing factors of commuting behavior from the perspective of resilience theory.

Variable			Dependent Variable			
			Commuting Space Pressure	Commuting Time Adaptability	Commuting Mode Adaptability	Commuting Resilience
Independent Variable			Exp (B)	Exp (B)	Exp (B)	Odds Ratio
Personal attributes	Type of jobs	Civil servant	2.37 *	0.56	0.33	1.20
		Enterprise staff	3.49 ***	0.43	0.11 **	1.02
		Self-employed laborer	0.36	1.89	0.13 *	0.58
		General service staff	Control group			
	BMI	Thin	0.38	1.15	0.11 **	0.30 ***
		Normal	0.48	0.97	0.67	0.55 **
		Slightly overweight	0.61	0.92	0.68	0.60 *
		Obese	Control group			
	Gender	Female	0.96	0.90	4.4 ***	1.71 ***
		Male	Control group			
	Age	30 and below	1.58	1.48	0.09 **	0.78
		30–40	0.93	1.40	0.39	0.81
		40–50	0.89	1.47	0.15 **	0.64
		50 and above	Reference group			
	Household registration	Non-local registration	0.58 *	0.95	2.51 *	0.85
		Local registration	Control group			
	Education level	High school degree and below	1.08	1.40	0.50	0.82
		Bachelor’s degree	1.04	1.07	1.32	1.09
		Master’s degree and above	Control group			
	Marital status	Unmarried	0.48 *	1.83	2.95	0.90
		Married	Control group			
	Number of family members living together	1	0.51	1.26	0.47	0.63
		2	1.27	0.86	1.15	1.10
		3	1.08	0.90	0.83	0.89
		4 and above	Control group			
	Personal monthly income	4000 and below	1.14	0.17 **	5.93 *	1.03
		4000–6000	0.98	0.19 **	2.00	0.94
		6000–8000	1.49	0.14 **	0.94	0.93
		8000–10,000	1.02	0.23 **	1.43	0.89
		10,000–15,000	0.67	0.25 **	2.94 *	0.90
		15,000 and above	Control group			
	Household monthly income	8000 and below	0.41	2.94	4.83	1.59
		8000–12,000	0.35 *	1.87	1.12	0.92
		12,000–16,000	0.42 *	1.98	1.75	1.23
		16,000–20,000	0.40 *	1.32	1.35	0.70
		20,000–30,000	0.81	0.65	0.86	0.86
		30,000 and above	Control group			
	Number of cars owned by family	0	1.03	0.16 **	84.57 ***	1.84 **
		1	0.90	0.36 **	5.56 ***	1.47 *
		2 and above	Control group			
Living and employment environment	Housing price in the place of residence		6.05 ***	0.49	0.05 ***	0.59
	Recruitment volume in the place of residence		1	1.00	1.03 **	1.00
	Diversity of infrastructure in the place of residence		0.44 *	1.60	4.98 ***	1.41 *
	Housing price in the place of employment		0.98	1.08	1.5 *	1.17 *
	Recruitment volume in the place of employment		1.00	1.00	1.01 *	1.00
	Diversity of infrastructure in the place of employment		1.04	1.08	0.89	0.95
Commuting environment	Subway station density in the place of residence		0.09 ***	2.46	19.3 ***	1.50
	Bus station density in the place of residence		0.91	0.93	0.74 ***	0.85 ***
	Distance from the place of residence to the city center		0.75 ***	1.19	1.43 **	1.07
	Subway station density in the place of employment		1.52	0.65	1.32	1.03
	Bus station density in the place of employment		1.11 *	0.99	1.16 *	1.09 **
	Distance from the place of employment to the city center		1.1 ***	0.93 ***	0.99	0.99
N			468	468	468	468
Pseudo R^2^			0.20	0.20	0.46	0.15
Log likelihood			–257	–190	–134	–292
LR Chi^2^			131	93	236	100.3
Prob > Chi^2^			0	0	0	0

*, ** and *** represent significant levels of 10%, 5%, and 1%, respectively.
